# Influences on Food Choices of Cambodian Women for Themselves and Their Families

**DOI:** 10.1177/2752535X251374278

**Published:** 2025-09-10

**Authors:** Janelle L. Windus, Kerith Duncanson, Tracy L. Burrows, Megan E. Rollo, Clare E. Collins

**Affiliations:** 1 5982 College of Health, Medicine and Wellbeing, University of Newcastle, Callaghan, NSW, Australia; 2 1649 Faculty of Health Sciences, Curtin University, Bentley, WA, Australia

**Keywords:** food choice, qualitative research, community engagement, Cambodia, women and children, Khmer

## Abstract

**Background:**

The nutritional status of Cambodian women and children remain poor despite implementation of dietary intake interventions. Cambodia-specific studies have explored how education influences nutrition and health behavior, but not factors influencing Cambodian women’s food decision-making for themselves and their families.

**Objective:**

To understand Cambodian women’s food decision-making, particularly related to barriers and enablers of healthy eating for themselves and their families.

**Methods:**

Qualitative descriptive methodology within an experiential paradigm involving four focus groups in rural and urban locations of Siem Reap province, Cambodia. Participants were Cambodian women aged from 18 years with at least one child under 5 years of age, and primarily responsible for food provision in their family. Data was analyzed using reflexive thematic analysis.

**Results:**

The three main themes identified were: (1) access impacts food choice; (2) responsible, nurturing food provision role; and (3) dichotomous perceptions of food. Access to food strongly influenced Cambodian women’s food choices, particularly cost and availability. Cambodian women were driven to fulfil their role and nurturing instincts to provide for their family within their capabilities and knowledge. They considered healthy food using a wide range of factors outside of nutrient values, including food texture, immediate body response, cultural food taboos and use of chemicals such as pesticides.

**Conclusions:**

To optimize nutrient intakes, practical, culturally appropriate nutrition interventions that address food access and perceptions of Cambodian mothers regarding their nurturing food provision role are needed.

## Introduction

Despite recent economic growth and reclassification as a lower-middle income country since 2015, Cambodia remains the second lowest income South-east Asian country.^1^ Cambodian children experience sub-optimal nutritional status with about one in five with stunting (22%) or underweight (16%).^2^ Cambodian women of reproductive age exhibit the double burden of malnutrition with 44% having anaemia,^3^ and rapidly rising rates of overweight/obesity affecting 33% of women aged 20 to 49 years.^2^

With 95% of Cambodians identifying as Khmer, the term ‘Khmer’ generally refers to Cambodian people, their native language, and their traditional foods. Cambodian women are taught gender norms through the centuries-old tradition of ‘chbab srey’; a list of rules that instruct Cambodian girls on the moral conduct, good manners and appropriate behaviour of a ‘proper woman’.^4^ Despite concerns of its impact on gender equality and gender-based violence, women believe following ‘chbab srey’ maintains their rich cultural identity as Khmer women, including instructions on domestic tasks, quiet demeanour and subservience to husbands. Within the defined Khmer gender roles, married Cambodian women provide food for the household and make associated financial decisions on their family’s behalf. Cambodian societal bonds are strong, with neighbours considered like family, and multi-generational households communally eating meals together from shared bowls of soup, spooning it onto their own plates piled with white rice.

Over the past two decades, studies in Cambodia assessing the efficacy of nutrient supplementation, food fortification, and home food production to enhance the nutritional status of women and children have reported varying levels of success.^5^ Cambodia’s recent demographic health survey^2^ reported only 57% of Cambodian women and 49% of children aged 6 - 23 months have dietary intakes that achieve minimum dietary diversity,^6^ indicating low nutritional adequacy. Dietary assessment studies that measured individual-level food and nutrient intakes of Cambodian women and children identified inadequate nutrient intakes, particularly vitamins A, C, thiamine and also zinc and calcium.^7^ With one in two Cambodian women experiencing food insecurity,^8^ the population is likely to experience ongoing challenges in providing healthy and nutritionally adequate meals for themselves and their families.

Few studies have assessed the nutritional knowledge of Cambodian women in the community,^5^ however, it is predicted to be low given the lack of nutrition information in school curriculums.^9^ Cambodian women may receive healthy eating advice from government health centres during ante- or post-natal appointments (Chea, M.; personal communication, 2024), or in selected communities through involvement with health programs run by non-government organisations.^10,11^ There are currently no country-wide food-based guidelines for Cambodians. One pilot program targeting school-aged children promoted a food pyramid for healthy growth and development^12^ and a USAID-funded project promoting healthy growth food groups depicting ‘energy’ ‘body-building’ and ‘protective’ foods, displayed in some health centres and hospitals.^13^ Ang et al. (2024)^14^ identified a notable gap in nutrition knowledge and awareness when exploring barriers to nutritious food consumption in two rural provinces. However, it is unclear whether nutrition knowledge informs or motivates the food choices that Cambodian women make for themselves or their families.

In promoting population health it is vital to understand food-related decision making, which is driven broadly by individual preferences, food environments and availability.^15^ According to Antin & Hunt (2012),^16^ studies promoting dietary change assume that people making food choices prioritise health, and consequently programs focus on increasing nutrition knowledge. However this premise overlooks the multi-dimensional and complex influences of food choices currently experienced in the Cambodian population, including implicit, unconscious and cognitive influences.^17^ For example, one study found Cambodian women expressed a fear of chemicals, defined as both known and unknown foreign additives perceived to adulterate food, particularly pesticides used on fresh produce in local urban markets, which were believed to harm their health, hence influencing their purchase decisions.^18^ Improved understanding of food choice influences may inform targeted intervention strategies that promote healthier dietary changes and result in improved nutritional status.

Therefore, this Cambodian community engagement study aimed to understand the motivators underlying Cambodian women’s food choices for themselves, their children and families, focusing on exploring perceived barriers and enablers of healthy eating decisions.

## Methods

The COnsolidated criteria for REporting Qualitative research (CoreQ) checklist was used to guide reporting of this study.^19^ Ethics was reviewed and approved by the University of Newcastle Human Research Ethics Administration (number H-2023-0226) and the National Ethics Committee of Health Research of Cambodia (number 362). Permission was also obtained from the provincial health department in Siem Reap.

### Study Design

The qualitative methodology was framed in an experiential paradigm^20^ focusing on exploring participants’ lived experiences, identifying perspectives and practices related to Cambodian women’s experiences making food choices for themselves and their families. Related to this experiential approach was the reflective theory of language which assumes participants’ thoughts, feelings and beliefs are communicated verbally in a straight-forward way, where the meaning of social norms and expectations were shaped by individual’s experiences from a realist perspective.^20^ Inductive analysis allowed the analysis to be driven by the data content.

### Participants and Recruitment

Participants were Cambodian women (18+ years) who were the mother of at least one child less than 5 years old who lived with them, and who provided food for the family through shopping and cooking meals. Participants needed to participate in focus groups facilitated in English with translation into Khmer language.

Cambodian women were recruited from Siem Reap city (urban) and Srey Noy (rural) in Siem Reap province. Using purposive sampling, a Cambodian research assistant in each location distributed information flyers to women around schools, preschools, neighbourhoods, workplaces and churches advertising the focus groups. Interested women contacted researchers for more information and eligibility screening was completed. Once verbal consent was obtained, women were invited to participate in a focus group and provided with venue details. Written consent was later collected from participants on arrival at the focus group. Participants were encouraged to share the flyer with other eligible friends and relatives. Research assistants made reminder phone calls the day prior to focus groups to confirm their intentions to attend.

### Methods

Four focus groups (in person) were conducted in February 2024 in Siem Reap province; two in urban and two in rural locations, with between eight and 10 participants per group, held privately in an open common area of a traditional Khmer-style house. Prior to each focus group a Cambodian research assistant collected demographic information from each participant.

After introductions and explaining the format, a focus group guide (Supplemental file 1) was used to generate discussion about perceptions of food in relation to health, and how they decide which food or meals to provide or not provide to their families. A series of five ‘conversation cards’ were then presented one at a time. Each card related to a key nutrient (vitamin A, thiamine, calcium, zinc and iron) identified from our previous research^21^ and showed images of food sources on one side and photos depicting the benefits of consuming adequate amounts of the nutrient on the reverse side (Supplemental file 1). The women were invited to share how they might consider incorporating these foods/ingredients into their family’s diet or recipes. Finally, three nutritionally enhanced Khmer mixed dishes were served with white rice, one at a time, and participants shared their opinions about the acceptability of each dish to themselves and for their families, contributing ideas for nutritionally enhancing similar meals they cook at home. Supplemental file 2 describes six trialled nutritionally enhanced versions of common Khmer mixed dish recipes.

JW asked questions in English, while a female Cambodian interpreter (RS) translated between English and Khmer languages. This ensured that JW, who can speak Khmer, could focus on facilitating and understanding the discussion and English first language researchers (JW, KD, CR) and Khmer participants could follow and contribute to the discussion. JW is an Australian PhD candidate who has lived in Cambodia for 7 years (2012 to 2019), KD is an experienced qualitative researcher from Australia, and CR is an Australian social worker who has lived in Cambodia since 2009. KD and CR captured observations in written field notes and debriefed with JW between focus groups.

Voice-recorders and smartphone videos recorded each 90-min focus group, enabling the interpreter (RS) to transcribe them verbatim into English using Microsoft Word, which were checked by JW. At the end of each group, participants were offered to review, modify or remove any comments, however all declined. Each participant was provided with USD$10 as reimbursement for their time and travel.

### Data Analysis

Reflexive thematic analysis (RTA) was used to analyse data due to its strength in identifying patterns of meaning from the dataset, as we anticipated Cambodian mothers would exhibit some similar perceptions and mindsets about food and meal choices yet potentially differ from each other in degrees of their influence and openness. The flexibility of RTA enables its application to the specific context of the Cambodian environment, which extended to exploring differences between urban and rural women’s experiences.

Consistent with Braun and Clarke’s (2022)^20^ six-phase approach to RTA, researchers JW and KD independently read and re-read the transcripts to familiarise themselves with the data, making detailed notes. Primary researcher (JW) then coded the dataset, identifying interesting and meaningful ideas relevant to the research question. Most of the initially generated codes had semantic meaning, while few were implicit. KD read, checked and discussed initial coding with JW, who then reviewed the codes, labelled with descriptors to capture their core meaning, and then grouped those with similar meanings. Themes and sub-themes were identified from the codes with shared meanings, initially generating eight candidate themes related to factors that impact the food choices that Cambodian women make. Candidate themes were individually described, differentiated and refined, resulting in three themes. Through discussion these three themes were defined by their characteristics and their names refined, then sub-themes were refined that aligned to each theme. Final descriptions of these themes and sub-themes were then shared with co-authors for the purposes of data triangulation and rigour, including presenting with quotes that demonstrated and reflected each core concept.

English-translated comments made by Cambodian women in the focus groups have been used to illustrate analytic points, with some quotes edited to remove unnecessary detail, signalled by [...] in the excerpt.

### Theoretical Perspective, Rigour and Researcher Positionality

This study assumed a critical realism ontological position^20^ whereby we, as accepted outsiders, sought to understand the experiences of Cambodian women positioned within their cultural context. The epistemological position closely fits with ‘contextualism’ by considering the unique context in which participants were situated that contributed to their knowledge, behaviour and perspective, which influenced the phenomenon of the study.

Approaches to ensure rigour in the analysis included the research team discussions between focus groups, double coding data, journaling, and triangulation of perspectives to increase the validity of findings, to build a more comprehensive understanding of women’s experiences.

Researcher backgrounds and positions influence our approach and analysis of this study.^22^ As primary researcher, I (JW) considered my position as an educated, middle-aged white Australian woman who lived in Cambodia for 7 years. I commenced a PhD (Nutrition) to identify culturally acceptable ways to optimise the health behaviours and nutritional status of Cambodians, also completing a qualitative research methods course. Researcher KD holds a PhD in nutrition and is an experienced qualitative and quantitative researcher, including previous diet-related research experience in Cambodia.

## Results

Thirty-four participants were involved in the four focus groups (18 rural and 16 urban women). No participants withdrew. Participants were aged between 24 and 46 years (mean 35 years), with a mean of 2.6 children (ranging from 1 to 6), and 3.5 people living in their households. Most women (*n* = 30) reported being married, with half (*n* = 16) indicating home duties as their occupation, 12 as farmers, and five worked for an organisation. Eleven participants achieved primary school education, 13 secondary school level and two had received tertiary qualifications, five rural women indicated never attending school. All participants indicated being involved in shopping or gathering ingredients and cooking meals for the household. Supplemental file 3 presents a table of participant characteristics.

Three themes were identified in the data: (1) food access impacts choice; (2) responsible nurturing food provision role; and (3) dichotomous perceptions of food. Each of these are presented in Table 1 and described below.

**Table 1.** Major Themes and Corresponding Sub-themes Illustrating Motivations of Food Choice by Cambodian Women for Themselves and Their Families (*n* = 34).

Theme 1: Food Access Impacts Choice	Theme 2: Responsible Nurturing Food Provision Role	Theme 3: Dichotomous Perceptions of Food
Characterised by the ability for Cambodian women to access food, ingredients and drinks according to their availability, cost and acceptability. Adequacy of foods impacts how food is accessed	Characterised by Cambodian women’s maternal nurturing instincts and responsibility to provide the best food/meals for her children and family within her capabilities and knowledge	Characterised by the different ways Cambodian women perceive the health-related qualities of food as opposing distinctive paradigms
Sub-themes		
Availability of food and ingredients	Providing nourishing meals	Wet vs. Dry
	Prioritising husband & children	Hot vs. Cold
Cost of food	Creatively responding to challenges	Helpful vs. Harmful
Acceptable food	Rewards of nurturing	Organic vs. Chemical
	Teaching/Modelling role	Khmer vs. Western foods
Adequate food intake	Relying on others to provide	Khmer traditions vs. Scientific (nutrition) knowledge
		Short-term vs. Long-term response

### Theme 1: Food Access Impacts Choice

Cambodian women described the substantial impact food access had on their decision making, including what was available, accessible, affordable and acceptable to them, reflecting their basic need for survival. Awareness about food availability and its nutritional value was also influential, as was adequacy of foods related to perceived dietary requirements of their children and other household members.

#### Availability of Food and Ingredients

Cambodian women described choosing foods and ingredients that are readily available for cooking or eating. While most women referred to shopping at the local produce market, they also described obtaining food and ingredients by foraging in ponds, forests and rice paddies, collecting water plants, fish, snails, frogs, mussels, wild potatoes and edible leafy greens. Women, particularly rural, shared many foraging experiences, indicating easy accessibility.

Sometimes I don’t have enough money to buy vegetables, but I found some vegetables grow around my gate and we don’t have money to buy some meat, but my house is near the rice field, and we could grab some crabs to cook. (P7, rural)

Growing vegetables and rearing livestock were also described as a source of food or ingredients, more so by rural women with accessible land.

I love to grow my own vegetables as I am living in the area of the […] mountain, I can pick some leaves or plant over there. (P5, rural)

Sometimes I ran out of money I went to my sister’s house and get some eggs there and cook fried eggs for my kids, and if I ran out of food I killed my own chicken and cook chicken soup. (P3, rural)

Both rural and urban women described picking fruits in season from trees within the community.

If I can go to the village I pick some banana [...] but sometimes my relatives come to town so they bring some for my kids. (P27, urban)

#### Cost of Food

Money was described as a key factor for Cambodian women when choosing food, for example, not consuming milk despite knowing it is healthy due to its high cost. Women explained how experiencing low funds reduced food choice, low variety or repetitive meals, and resulted in choosing less preferred options such as dried meat instead of fresh meat.

If we can’t have money to buy vegetables, I go look for morning glory in the field. (P3, rural)

If I cook for the people I love, I need to think about the budget first. If we have enough money, yes I can cook better food; I don’t usually choose to cook with dried meat or fish. (P5, rural)

For my family we cook about the same style of food because we don’t have much money. (P5, rural)

Some women described making lower-cost compromises which resulted in them consuming poorer quality and/or nutrient-poor food options.

Milk and yogurt for my children it is not possible [...] so I choose coconut water and milk in the can. (P33, urban)

Women experiencing low affordability were prompted to seek alternative ways of sourcing food, such as foraging or obtaining food ingredients from extended family or neighbours.

This season we can find brij [edible leafy greens] leaves and we can cook it with fish, but if we have money we can buy some pork rib and make soup with brij leaves. (P5, rural)

#### Acceptable Food

Cambodian women indicated taste was a critical factor in choosing food, prompting them to provide delicious meals for their family to gain their approval.

I didn’t even realize that I can cook in different styles and dishes with good flavour. Now my husband can eat my food and not complain as before. (P33, urban)

The significance of white rice for Cambodians was clearly expressed, such that they feel they haven’t eaten if a meal does not include rice. Colloquially to ask a Cambodian if they have eaten a meal is ‘have you eaten rice already or not?’.

We just eat what we have, as long as we have rice it is our luck. [...] we don’t have money to buy any snack just only rice, that cause us tired. (P7, rural)

It is what my husband likes and they can have some rice which is left over last night for breakfast with some broth before they go to school. (P9, rural)

This became more apparent when discussing brown rice, which most participants had never tried, nor did they want to. Those who had tried it strongly disliked the flavour and firmer texture, despite learning of its health benefits. Cambodian women referred to foods that were considered unacceptable to both adults and children as not knowing how to eat them.

It is hard to eat brown rice [...] the flavour is different, hard to chew and digest, that’s why most of Cambodia choose white rice. They don’t know how to eat it. (FG3, urban)

#### Adequate Food Intake

Women referred to consuming adequate amounts of food to satiate themselves and their family from hunger.

I think it makes [us] tired because we work hard but we cannot eat enough food with nutrition [...]. We sometimes eat salt and chilli with rice to fill our stomach. (P7, rural)

They described consuming healthy foods to support body and brain growth in their children, strength for physically hard-working husbands and energy to complete their daily chores.

Why I choose grosang teap [edible leafy greens]? Because it has lots of nutrition for Khmer people, and beef is good for protein and the young people need more meat to help their body as well. (P33, urban)

Some women mentioned the importance of breakfast for children so that they can concentrate at school, providing three meals a day to ensure family receives enough food, and for the pregnant woman in the study eating enough to provide for her unborn baby.

They [children] need some food in the morning otherwise they get stomach pain, and then they go for bath and they have some energy. (P9, rural)

### Theme 2: Responsible Nurturing Food Provision Role

Cambodian women’s nurturing nature and cultural expectations motivated them to provide food for their children and family, which they believed was their role as responsible mothers.

#### Providing Nourishing Meals

Women expressed their desire to nurture their children through providing a variety of healthy, delicious meals that they believed would meet their children’s nutritional needs for growth, indicating this is what responsible mothers do.

I don’t follow what they want to eat. When I make soup, they don’t eat the meat, but they drink the broth, some vegetable and eat it with rice. (P11, rural)

These women also shared their desire to provide food they believed their husbands needed to nourish their bodies so they could work effectively and energetically.

For my husband needs more soup for his meal because broth could give him nutrition and strength for working. He lifts some big wood in the forest and samlor is good. (P8, rural)

Ultimately, these Cambodian mothers made food decisions for their families to the best of their ability within their capacity, based on what they considered to be healthy foods.

I have to be responsible to cook for them in my house [...] because of that I choose vegetables (P33, urban)

#### Prioritising Husband and Children

It was evident that Cambodian women prioritised their husband and children’s nutrition needs and food preferences ahead of their own. Some women indicated their first priority was their husband, choosing to cook and serve the meals he likes in order to please him.

So, it was really hard to decide, but I followed the head of the house. So I make vegetable soup [...] because my kids only wanted to eat dry food, but the husband is the opposite, he likes soup. (P11, rural)

Other women prioritised their children’s needs and preferences over their own.

For the food I choose to make soup. Before I do not like soup but after we had children we change according to the kids. (P32, urban)

Women reported only consuming foods they personally preferred if they were alone or away from family.

I really like spicy food, but my household, husband and kids cannot eat it, so I cannot eat it either. (P17, rural)

Women’s decisions-making process considered how they prioritise their family’s food preferences and their own nutrition knowledge.

I want them to eat vegetables [...] so I include them as much as possible, but my family does not like it. I try to cook with my best as I can cook for them because when I eat vegetable my body can fight with disease so they can protect their healthy body too. (P22, urban)

#### Creatively Responding to Challenges

Women described striving to provide appealing meals within context of their unique circumstances, such as lack of money, cooking ability, lack of time available, or their household composition.

When I cook food, I choose one dish that we all can eat all together, which the little kids and the adult can eat too. (P13, rural)

Participants described creative ways to address their unique challenges. For example, one woman was motivated to cook two dishes to cater for the preferences and needs of all household members. An urban woman mentioned her children’s rejection of vegetables, so she would explore social media to find new non-traditional ideas or dishes for including vegetables, such as savoury fried vegetable cakes.

My kids are picky with some vegetables. If I make soup they won’t eat [...] so I make as a cake, using vegetables such as carrot, pumpkin, potato [...], mix with some meat and deep fry as cake so she might eat vegetables because I watch YouTube. (P32, urban)

#### Rewards of Nurturing

Cambodian women shared how satisfying it was for them to provide adequate, delicious meals for their household, expressing this with a sense of pride.

I need to cook to please my husband and most of time I can take some rice with soup broth for my little kids. My husband loves that way of cooking. (P8, rural)

If I have time I will make some desserts once or twice per week, and sometimes my kids and my husband give me thumbs up. (P7, rural)

Women expressed delight in their family enjoying the healthy food they provided for them.

And when we see our kids have good snack and happy about their snacks, I am happy to see my children grow in healthy way. (P29, urban)

#### Teaching/Modelling Role

Some mothers described examples of healthy food options, such as including a variety of vegetables at every meal, choosing grilled meat over fried, and offering fruit instead of fruit drinks or fried fruit snacks.

The snacks that I always have for my kids and family daily are banana, papaya and oranges. And the thing that I don’t want them to eat is deep fried banana. It is oily and not good for their health. (P21, urban)

Women perceived these practices as a teaching opportunity and a model of healthy eating behaviours to their children. Several women described intentionally including different vegetables in soups to increase children’s exposure to them.

I love to teach them learning how to eat vegetables. Every day they can eat most of vegetables except bitter melon. (P21, urban)

#### Relying on Others to Provide

Working mothers expressed regret or guilt for not personally providing all meals for their family, regularly relying on the child’s father or grandmother to provide meals. They indicated how this often resulted in less healthy meals being provided to their children, as fathers choose to serve food that they preferred and is easy to cook.

From Monday to Friday, my children are with my husband, and he loves to eat something fermented. (P33, urban)

Working women described feeling the need to ‘make up’ for the lack of healthy foods, particularly nutrient-rich vegetables, after their children have been fed by others.

There are only two days that I can choose food to cook for them so I have to choose that kind of dish [soup with vegetables] because she [daughter] didn’t have a chance to eat more vegetables.” (P33, urban)

According to Cambodian women, other family members tended to give their children unhealthy snacks, while the mother usually provided them with fruit, or in a few cases, milk.

As a mother we never buy packet snacks for them, but sometimes when we are away, other people or members in the family were sneaky and give to them. (FG3, urban)

### Theme 3: Dichotomous Perceptions of Food

The health-related qualities, features of, and responses to foods were perceived by Cambodian women in a highly dichotomous manner.

#### Wet versus Dry Food

Cambodian women referred to wet foods, i.e., soups and samlors (thick soup) which they perceived to have healthy traits because they included a variety of vegetables and meat/fish in one dish.

I want my kids to have some broth to eat with rice, and this soup [...] we can put carrot, radish and we can put some chopped pork or pork ribs and some livers to gain more nutrition, so when we eat we feel we get energy [...], strength and good health. (P21, urban)

The soft, moist consistency of ‘wet’ foods were described as more suitable for young children to eat more easily. They considered the soup broth by itself to be rich in nutrients and energy to meet children’s and husbands’ requirements for study and work.

The broth came from the nutrition of meat and vegetables, and all the nutrition stores in the broth, and when you eat broth with rice it helps us swallow easily, (P23, urban)

Women also referred to ‘dry’ foods as fried or grilled meat/fish or raw vegetables, describing them as more difficult to eat, particularly for children. They explained serving dry foods with a dipping sauce or juicy fruit adds necessary moisture to aid chewing and swallowing.

If I choose to grill fish or meat I have to choose watermelon or mango to eat with them (P33, urban)

#### Hot versus Cold Food

Women described foods that make their body feel hot, such as chilli or ginger, which combats sensations of coolness, and can either bring healing or cause sickness.

The food that they like is fried ginger chicken. Ginger helps the stomach and fighting the cold and of course they love it too. (P34, urban)

The old ages said any food you put more black pepper it helps our veins in body strong and help our womb too. […]. It keeps the heat in our body. (P31, urban)

Conversely, they described foods that are cooling to their body or stomach, like vegetable soup, which can overcome hot body sensations.

When you eat different kinds of vegetables it helps your stomach feel cool, and good for your intestine when you go to the bathroom. (P23, urban)

#### Helpful versus Harmful Food

Women perceived helpful foods to be those with health benefits, giving examples such as vegetables fighting disease, soup broth giving energy, meat providing strength, and fruit promoting healing.

The food that help me when I am weak and exhausted is soup, like beef soup (P17, rural)

For about vitamin of passionfruit, it has a lot of vitamin C and it helps fighting the cold and recover from the cold. (P29, urban)

Food considered to be harmful, and therefore unhealthy were those reported to cause symptoms of intolerance or allergies, such as rashes, breathing problems and bloating. Examples mentioned included fermented foods or spicy foods.

I can’t eat anything fermented because after I eat I feel headache, sore throat and get stuffed nose and get cold easily. (P32, urban)

I also like spicy food, [but] it causes me stomach pain (P15, rural)

Women consistently reported the belief that combining unfamiliar or certain green leafy vegetables in soups was poisonous to their bodies.

We thought and follow the rumour that if we cook more [green leafy] vegetables it will harm our body or health or cause food poisoning. (P21, urban)

Women also reported the dangers of sweetened and energy drinks, especially causing harm to children.

We are not allowed to drink energy drinks or any other green tea [Oichi] and any sweet drinks […]. For the children, they are not allowed to drink energy drink, it harms the brain. (P31, urban)

#### Organic versus Chemical

Use of chemicals, such as pesticides in fresh produce was a consistent concern expressed by these Cambodian women. They considered organic fresh produce to be absent of chemicals and therefore better for health.

I won’t buy any vegetables from the market at all. There are some ivy gourd leaves as well so we can pick some and cook it, [...] so I normally eat organic vegetable to make my body healthy. (P5, rural)

Cambodian women considered vegetables sold at local markets, particularly in urban areas, to contain harmful chemicals and hence considered them less healthy. They indicated organic fresh produce was sourced from small family farms in rural villages which they preferred to buy, or grow their own organic vegetables.

When we see the villagers come to sell the green leaves and vegetables, we often buy from them [...]. But the vegetables that sell on the market nearby I don’t usually buy it [...] they use too much chemical, it might cause us stomach pain or food poisoning, diarrhea. (P30, urban)

It is good to grow vegetable by ourselves. It is organic. (P4, rural)

#### Khmer versus Western Food

Cambodian women expressed the belief that specific traditional Khmer soups or samlors are very healthy because they contain a variety of vegetable types, meat or fish, and are easy to eat for all household members.

I think [samlor] koko and brohour is healthy because it contains different kinds of vegetables (P14, rural)

Healthy food is combined with meat, a lot of green vegetables, fish, morning glory, water lily, […], and the things that are vegetables […] make us healthy. (P12, rural)

Women shared experiences of learning to cook traditional Khmer meals from family members (mothers, uncles) or from observing chefs at festivals. They described how they were expected to learn to cook while young to fulfil their future role as wife and mother.

When I was growing and [...] the feast we have in the village or attended a ceremony [...] we like cooking so we watch them and now I can cook anything. (P32, urban)

I have learned how to cook from my mother. As the old ages saying ‘if the woman doesn’t know all about the kitchen stuff she won’t able to be ready for marriage’. (P34, urban)

The dichotomy of this sub-theme was highlighted by comments from urban Cambodian women who indicated how Western food has begun influencing them and their family. The women proudly described ways they had learned to cook western dishes, such as pasta dishes, predominantly through social media or their Western employer, and how their children are beginning to request western style meals and snacks.

For me if I cook western food I have to watch YouTube. (P29, urban)

She [daughter] likes some food as Westerner for her snacks. She likes to eat pizza, burger and sandwich, and for the fruits she likes only the expensive ones. (P32, urban)

Presumably, this dichotomy of differing expectations of younger and older family members will become increasingly prominent and conflicting for Khmer women in the future.

#### Khmer Traditions versus Scientific (Nutrition) Knowledge

Some women exhibited a basic knowledge of nutrients in foods, referring to vitamins in vegetables, vitamin C in citrus fruit, protein in meat, and iron in morning glory. A few women recalled learning about three food groups (energy-foods, body-building foods, protective foods) at school or on posters displayed in their workplace or health centres.

I believe food that make us healthy is coming from the 3 groups of the meal […] protective food as vegetable, fish meat in building body food group, and energy food has some corn, potatoes, palm sugar, and eat with organic vegetable; it helps [make] you strong.” (P2, rural)

Some women indicated they had limited knowledge about the nutritional value of foods.

Never known before about the benefit of brown rice (FG3, urban)

Awareness of unhealthy foods predominantly referred to foods high in sugar, or for a few women salty or oily deep-fried foods.

I love to drink avocado shake but for fresh fruits I will eat more than the drinks or fruit shake or soft drinks because they give more sugar. (P32, urban)

The drinks that harm our health are the drinks stored in the can, such as coke, energy drinks [...] and they don’t want us to cook with too much salt or too much sugar, it is not good. (P31, urban)

Cambodian women explained how Khmer traditions and cultural beliefs influence their food choices, particularly during pregnancy and breastfeeding having been forbidden to eat spicy foods, fermented foods and certain specific foods such as clams and bamboo shoots.

When I was pregnant, I’m not allowed to eat spicy food such as papaya salad, dried clams, deep-fried meatballs, fermented food […] it harms my baby in the womb too. (P26, urban)

After I gave birth to my baby, I wanted to eat bamboo shoot but my mother said I could not eat it; it harms my womb, ovary and health. And some chilli or spicy food or any sour fruits I could not eat either; if I eat it makes my baby get trouble or I get diarrhea too. (P22, urban)

### Short versus Long-Term Response to Food

Cambodian women related how eating foods made them feel, predominantly describing immediate or short-term effects. They perceived foods that induced instant symptoms of intolerance or allergy to be unhealthy, while healthy foods were ones that made them feel good and were associated with giving energy and strength.

I will get diarrhea, any different kinds of fermented fishes I can’t eat it. Whenever I eat I get headache right away. (P2, rural)

It [green leafy vegetables] feels good on my stomach and especially it is good for our intestine as well. (P20, urban)

Very few women expressed concern regarding long-term effects of foods related to the risk of developing noncommunicable diseases (NCD), while those who did mentioned the causes were excessive salt or sugar.

If we eat too much sugar it causes us diabetes [...]. We are advised to eat something that is just plain. (P31, urban)

When prompted, a few women mentioned adjusting sugar for a family member diagnosed with diabetes or reducing salty foods for hypertension.

I won’t choose to cook food with too much sugar such as Kor [sweet stew] because my husband has diabetes, so I have to avoid some food with sugar, but use fruits as natural to have sweet taste instead. (P33, urban)

If you found yourself with diabetes you have to stop eating the fermented food. (P30, urban)

### Cambodian Women’s Responses to Modifying Their Traditional Khmer Mixed Dishes

Initially, Cambodian women expressed a cautious scepticism about modifying their traditional mixed dishes, with resistance to changing their usual recipes and risking judgement from others of their cooking ability.

They would wonder where did you learn that? Why you put the garlic chives in? Who taught you how to cook? (FG4, urban)

After being presented with each mixed dish, they spent considerable time examining it, curiously discussing between themselves how atypical and ‘weird’ these ingredients were for each Khmer mixed dish. They voiced distrust of the dish, expecting it would be unacceptable.

It is weird. For soup normally I put herbs, fish, and mushroom, and never put other green leaves [moringa]. (P14, rural)

If you said to put the agati leaves, but they don’t have time to taste your dish first, they don’t feel trust. (P22, urban)

Once the women tasted each dish, they expressed with surprise and delight that the dishes were tasty and quite acceptable, confirmed through observing them eat a good amount of each dish. Women described these modified recipes as ‘unique’ or ‘creative’, and many declared they would like to try this dish with their family at home, expecting their husbands, and for some their children would enjoy it, and benefit from the extra nutritional value.

I used to eat local sour soup before, but the flavour is okay. Yet at the time I ate your sour soup is very unique, so yummy. All 3 dishes you cooked are so delicious and I love to copy it and make it for my husband. I learn a lot. (P3, rural)

Women acknowledged the added ingredients to these dishes were familiar to them, and being common, accessible ingredients made these recipes even more appealing and feasible to try.

When I go back home I will try to make this […] now I can add more vegetables. At my house we have moringa […], I will try, if it will turn out yummy like here. (P12, rural)

However not every woman expressed a positive response of the new recipes, being too unusual for some.

The food I tasted, it is very strange, all the three dishes (P7, rural)

## Discussion

The current study identified three major themes related to making food choices: ‘food access impacts choice’, ‘responsible nurturing food provision role’ and the ‘dichotomous perceptions of food’. Themes one and two align with previous research among populations in other low-income contexts, especially related to food access and availability, cost of food, and a mother’s desire to provide healthy food for her family.^15,23–25^ Conversely, theme three is unique to the Cambodian context as it depicts a variety of ways Cambodian women considered food influences health, such as meal texture or composition (wet or dry), reaction when consumed (helpful or harmful), and chemicals (present or absent, i.e., organic). Although there were some notable differences between rural and urban women’s food-related decision making such as more foraging in rural areas, buying chemical-free produce from rural markets, and urban women seeking western dish recipes that are presented in the results, these were not considered dichotomous internal drivers of food choice.

These findings align with Saurman et al’s adapted Theory of Access (2015)^26^ referring to users’ access to health services, specifically the six dimensions cited as integral to access: accessibility; availability; acceptability; affordability; adequacy and awareness. The themes and sub-themes from the current study align with Saurman’s dimensions, namely ‘food access impacts choice’; ‘availability of food and ingredients’; ‘acceptable food’; ‘cost of food’; ‘adequate food intake’ and ‘Khmer traditions versus scientific knowledge’. This alignment supports the feasibility of these dimensions, implying that having nutritious food access is crucial for diet adequacy. Aligning strategies to these sub-themes may increase access to food for Cambodian women to choose from, with further research in Cambodia required to explore efficacy of these strategies.

Cambodian women described approaches for addressing low affordability and accessibility, such as foraging for edible leafy greens and animal protein sources in ponds and fields or gathering ingredients from family or community members. The high cost of healthy food will continue to limit food choices, and lack of variety will likely impact on their nutritional status. Some women shared experiences of inadequate food intakes, having just enough rice to satisfy their hunger let alone provide nutritionally adequate meals. These findings align with previous Cambodian research where women reported that poor nutrition and a lack of food were due to a lack of money.^27^ Budget also impacted food acceptability as less-preferred, more affordable options were chosen, despite valuing their ability to provide satisfying, delicious meals for their family. Taste acceptability was a highly important factor most notably with the acceptable flavour of their staple food, white rice, and rejection of the healthier brown rice alternative.

This study adds to existing research reporting that Cambodian women consider food across a variety of criteria, influencing their view of the healthfulness and family food provision. Examples include considering wet food as being healthier than dry, their perceived healthiness of food related to making their stomach feel hot or cool, or avoiding chemical-laden vegetables or taboo foods during pregnancy and breastfeeding for the safety and health of mother or child. Two thirds of postpartum rural Cambodian women practice food taboos,^28^ which restrict food consumption and put their nutritional status at risk, particularly considering these women also prioritise their family’s needs over their own. Sensitively targeting these restrictive ideations, while encouraging consumption of nutrient-dense foods, is recommended for health promotional strategies with Cambodian women.

Cambodian women perceive the immediate effect on their body as depicting whether a food is harmful. For example, experiencing immediate physical reactions consistent with food intolerances or allergies is considered detrimental to health, whereas consuming soup/samlor broth is believed to provide immediate energy and strength required for hard physical work. While the women believed that combining certain green leafy vegetables makes them poisonous to eat, they readily consumed those foods in the focus group. Nutrition education strategies that empower women by aligning health-promoting, evidence-based nutrition messages with food beliefs were shown to be effective in this research when implemented using experiential approaches. It is feasible that healthful nutrient-rich versions of traditional Khmer dishes could be shared at individual and population levels in Cambodia.

The sense of guilt Cambodian women experienced when failing to provide optimal foods to their children is common across cultures and income levels, impacted by the ‘value conflicts’ of combining healthy food choices with their children’s taste preference.^29^ This study highlights that the women’s role was to provide adequate food, prioritize their family’s needs over their own, while aiming to teach and model healthy eating habits. This underscores the importance of ensuring their knowledge of healthy foods and dietary patterns is accurate, realistic, and culturally appropriate. While Cambodian women in this current study exhibited basic nutrition knowledge, it was often a lower priority in food choices. However, they were motivated to raise healthy children and open to learning about nutrition. Simple, targeted, practical messages about nutritional benefits may be more effective than a scientific approach in the Cambodian context.

Cambodian women did not express concern about the impact of food on their long-term health outcomes, which is congruent with survival behaviours many women exhibited such as daily market shopping and foraging for food due to low funds and poor storage options. While strong links between poor nutritional quality dietary intakes and NCDs have been recognised in high-income populations, the burden of NCDs is significantly higher in LMICs.^30,31^ This study found Cambodian women were considerably more concerned about consuming an adequate amount of food daily than long-term dietary risk, however there was some awareness of NCDs among Cambodian women when a family member had received a NCD diagnosis and they were required to manage it by serving ‘plain’ food, reducing sugar or salt, or avoiding fermented foods and sweet drinks.

The current study’s results demonstrate that Cambodian women were predominantly consuming traditional Khmer options, however the transitioning food environment that is increasingly evident in Cambodia^32,33^ is reflected in this study as urban women are choosing Western food options where they are widely promoted and available. Internet access^34^ and increasing use of smartphones by women^34^ makes seeking Western food recipes on social media easy. However, it is suggested that less nutritious Western foods are not necessarily replacing traditional options but are being eaten in addition to their regular intake.^33^ Similarly, sweetened beverages and commercially produced snack food consumption by young children has been increasing, driven by paternal food choices and poor healthy food perceptions.^32^ This Western food trend is consistent with the nutrition transition evident in most LMICs,^35^ where increasing access to Western food is congruent with rising rates of overweight, obesity and diabetes among Cambodian women.^2,36^ This is concerning as Cambodian women in this study were seemingly unaware of the negative long-term health effects of shifting from traditional to Western foods, typically high in sugar, salt and fat and highly processed food.^35^ Some Cambodian women feel conflicted between providing traditional Khmer meals and learning modern dishes. While they aspire to offer healthy options, there is a risk of choosing less healthy Western meals without proper knowledge. The higher cost of Western foods in this region is also a concern. Thus, culturally appropriate health promotions should prioritize raising awareness of dietary approaches to reduce NCD risk and provide effective dietary management education.

The study explored improving the Cambodian diet by trialling nutritionally enhanced traditional dishes. Initially, Cambodian women were sceptical, suggesting that mere promotion is ineffective. However, their positive response to the taste and nutritional benefits of modified dishes highlights the importance of personal engagement. Confidence to try new things grew within like-minded groups, reflecting their collectivistic culture. Further research should collaborate with communities on recipe enhancement trials to explore better strategies to influence healthy meal choices and increase nutritional intake.

## Limitations & Strengths

A key strength of the current study was the researchers’ extensive cultural and language understanding, gained from years of living in the Cambodian community. This facilitated the planning and logistics of the focus groups. The study built on previous research identifying common recipes and key nutrients lacking in the Cambodian diet. It involved Cambodian women from both rural and urban settings. Although data from other provinces might have provided different perspectives, the concepts discussed were consistent across groups. The use of purposive and snowball sampling, while suitable for qualitative focus groups, may have influenced the data collected.

### Implications for Research and Practice

• To help Cambodian women make healthier choices within their budget, consider these accessible options: (a) select nutrient-rich vegetables like dark green leafy vegetables over less nutrient-dense options like white cabbage at similar prices; (b) choose plain, unsweetened milk instead of sweetened milk products, including promoting the suitability of long-life milk; (c) replace packaged snacks and sweetened drinks with seasonal fresh fruits, vegetables, or lotus seeds.
• To effectively promote healthy eating, consider co-designing interactive, communal nutrition sessions that: (a) recognize how women perceive healthy foods; (b) sensitively address food taboos, chemicals, and long-term dietary impacts; (c) demonstrate healthy eating patterns and meals with community involvement; and (d) provide simple health messages and tools for women to teach their children and household members.
• To promote healthier food choices, facilitate community-led activities that encourage discussions and creation of modified or alternative healthy food options. Focus on sampling rather than just suggesting, such as through innovative cooking classes featuring nutritionally enhanced recipes or healthy Western-style foods.

This study explored the motivators of food choices among Cambodian women, identifying access to and perceptions about foods as strong influencers. It highlights the importance of understanding these perceptions to create targeted health promotion strategies. Cambodian women prioritize food texture and chemical-free over nutrition. To support their role in providing and modelling healthy eating, it is crucial to consider their beliefs and motivators and use experiential strategies to equip them with the necessary knowledge and tools.

### Descriptors of Khmer Mixed Dishes

Samlor (សម្ល) is a common descriptor for thick soup, with specific variants, usually made from fish or meat with one to two vegetables, e.g., samlor brohour (សម្លប្រហើរ), or samlor koko (សម្លកករ) which particularly contains a larger variety of vegetables. Grosang teap (ក្រសាំងទាប) is a rat ear leaf (Peperomia pelludica) similar to watercress. Brij (ស្លឹកព្រិច) are leaves from a Sweetleaf tree (Melientha suavis). Kor (ខ) is a sweet stew usually with pork or fish, traditionally using palm sugar.

## Supplemental Material

Supplemental Material - Influences on Food Choices of Cambodian Women for Themselves and Their Families Supplemental Material for Influences on Food Choices of Cambodian Women for Themselves and Their Families by Janelle L. Windus, Kerith Duncanson, Tracy L. Burrows, Megan E. Rollo, and Clare E. Collins in Community Health Equity Research & Policy.

Supplemental Material - Influences on Food Choices of Cambodian Women for Themselves and Their Families Supplemental Material for Influences on Food Choices of Cambodian Women for Themselves and Their Families by Janelle L. Windus, Kerith Duncanson, Tracy L. Burrows, Megan E. Rollo, and Clare E. Collins in Community Health Equity Research & Policy.

Supplemental Material - Influences on Food Choices of Cambodian Women for Themselves and Their Families Supplemental Material for Influences on Food Choices of Cambodian Women for Themselves and Their Families by Janelle L. Windus, Kerith Duncanson, Tracy L. Burrows, Megan E. Rollo, and Clare E. Collins in Community Health Equity Research & Policy.

## Data Availability

The datasets generated during and/or analyzed during the current study are available from the corresponding author on reasonable request.*
