# Supplementary material for: Influences on Food Choices of Cambodian Women for Themselves and Their Families
Source: Community Health Equity Res Policy. 2025 Sep 10;47(1):49–61. doi: 10.1177/2752535X251374278 (PMC13499906; doi:10.1177/2752535X251374278)

Supplementary File 1:

Cambodian women's focus group guide and nutrient cards

When participants arrive at the venue (Research Assistant to do):

- Welcome participants and check their name off the register
- Screen with screening form, if not completed already
- Invite them to sign the consent form
- Ask them to self-complete the demographic questionnaire; assist as required
- Ask if they prefer we use their own name or a pseudonym in the report we write, record on register
- Write their name / pseudonym on a sticker, place it on their chest
- Take a seat at the table or on the mat; give them a bottle of water

When you are ready to begin the group (Facilitator):

- Thank everyone for joining in
- Introduce yourself and the translator, saying *I'll lead the discussion, asking questions in English, and the translator will translate into Khmer. Then your responses will be translated into English.*
- Introduce other helpers by name
- Advise them where the bathroom is and to silence their mobile phones
- Describe what will happen in the group, time it will finish, answer any questions
- **Introduce topic:** *We are conducting these groups to learn about your perspective about health, and the foods you cook and serve to your family at home. We want to hear about your experiences and ideas – there are no right or wrong answers. We will also talk about the benefits of some nutrients, then share some Khmer food with you, and ask you to share your thoughts about these. We will use this information for developing healthy food programs with Cambodian communities*.
- *We encourage each of you to share your thoughts during the focus group. If you hear someone say something that isn't the same for you, please share your thoughts with the group. Please try not to interrupt each other, and always show respect for each other.*
- Remind participants they may revise or withdraw comments by notifying the facilitator by the end of the group; and withdraw from the project at any time without giving any reason
- Remind everyone that we will video and audio record the discussion.
- Discussion will take 1½ hours (finish at 11am \ 4pm)
- Ask everyone to state their name before they speak each time so they can be identified on the recording. Use first name or pseudonym only.

Any questions? Start recording now.

Focus group question guide:

Q#	Question	Prompts	Aim
1	*Icebreaker*: Please say your name, how many children you have, and your favourite meal	Remind people to say their name, and keep it brief	Get the participants talking, researchers to recognize names/voices
**Great, thank you. From now on, we won't go around the room for each answer. But we'll try to have more of an informal conversation. So, feel free to respond to what other people are saying**			
2	In what ways do food and drinks you consume make you	Eg, you mentioned eating ……, are there other foods that make	Understand perception of what makes people healthy;

<table>
<tr><td></td><td>healthy?
Or
In what ways has the food and drink you consume helped you feel healthy?</td><td>you healthy?
What is it about these foods that makes you healthy? Eg, vitamins? Iron?
Listen for…
Certain foods, specific nutrients, water, safe/clean food, enough food, eating patterns</td><td>the impact of diet on their health
Exploring their understanding of nutrients- health relationship</td></tr>
<tr><td>3</td><td>What foods/drinks do you avoid eating?
- Because of health?
- Other reasons?</td><td>Listen for…
Long-term health
Immediate reaction / short term feelings of wellbeing</td><td>How participants understand what makes them unhealthy; the role of diet on their lack of health</td></tr>
<tr><td>4</td><td>Think about foods that you want to eat but don’t. What is stopping you from eating foods that you want to eat?</td><td>Because of food taboos?
Listen for…..
Spiritual / Food taboos, cultural behaviours during pregnancy / lactation</td><td>Barriers/enablers to healthy diet</td></tr>
<tr><td>5</td><td>How do you decide what meals you cook and eat….
- for you?
- for your family?
Which foods/drinks that your children consume are different to those you consume?</td><td>Listen for…
Food/drink choices, environment, budget, healthy/nutritious options, safe/clean foods, easy access, someone else chooses</td><td>Motivators of food choice
The degree that nutrition/ health influences food choices</td></tr>
<tr><td>6</td><td>What about snacks? Which foods or drinks do you / your children eat for snacking?</td><td>Which snacks do your children eat that you prefer they didn’t eat?</td><td></td></tr>
<tr><td colspan="4">Thanks for talking about your diet – that’s really helpful.
Four years ago we collected information from Cambodian mothers and their young children about the food and drinks they ate. The results showed that Cambodian diets tend to be low in some important nutrients that our body needs to function well.
Let’s look at which foods provide some of these nutrients, and how eating enough will benefit our bodies, then I’ll ask you to share your thoughts. vitamin A, thiamine, iron, zinc, calcium
Show nutrient cards (side 1: pictures of high-source foods, side 2: pictures representing benefits of adequate intake), one at a time (random order)</td></tr>
<tr><td>7</td><td>Seeing these health benefits of (nutrient) and the foods that provide it, how could you include these foods in your diet, or use these foods in your meals?</td><td>If already consume…..Could you increase these foods in your diet/meals?
What could you swap (food) with?</td><td>Identifies how likely they are to adopt different foods/eating patterns
How motivating nutrition is in food choice</td></tr>
<tr><td colspan="4">Have Khmer dishes ready to serve. Give a plate of rice and spoon to each participant.
Exploring new ways to make your Khmer mixed dishes more nutritious, we have cooked 3 Khmer</td></tr>
</table>

**dishes. We will ask you to look at and taste each one and tell us what you think.** ***Present each dish one at a time, with rice.***			
8	Introduce each dish<br>Obtain initial reactions<br>This (name of dish) has (added ingredient) which provides (nutrients)<br>Or<br>We have increased the nutrient quality by adding/reducing/switching …….. in this dish.<br>What do you think of the look? taste?	What would members of your household think of this dish? children, husband, others.<br>How likely is it that you would make this modification to this dish at home?	Gauge acceptance/likelihood to adopt change<br>Explores how motivating nutrition is in changing food choices to healthier ones
9	What would you say to your sister or neighbour about what we have talked about today or about these recipes?	If positive, ask…In what ways can we share this information to your community?	Gauge whether this information is useful to the community and worth sharing.
**Thank you so much for your time, and for participating in answering these questions for me. I hope you enjoyed our group. Your responses have been recorded and if you have changed your mind about any responses you have made, please let us know now and we can remove these.**<br>**Obtain interest in participant receiving a graphical summary of this research once completed (record name).**			

Conclusion:

- Indicate on register if they would like to receive a short summary of this research (about 6 months)
- Sign register and receive incentive envelope

Khmer assistant to prepare room, prepare meals to serve (10:30am or 3:30pm)

Resources required: floor mat/table, consent forms, ink pad, participant incentive ($10 each), 3 x Khmer mixed dishes to sample, bowls, spoons/forks, plates of white rice, water bottles, napkins, 2x phones to video, audio recorder; 5 nutrient cards (calcium, zinc, vitamin A, iron, thiamine)

NUTRIENT CARDS

Slide 1: food sources of calcium

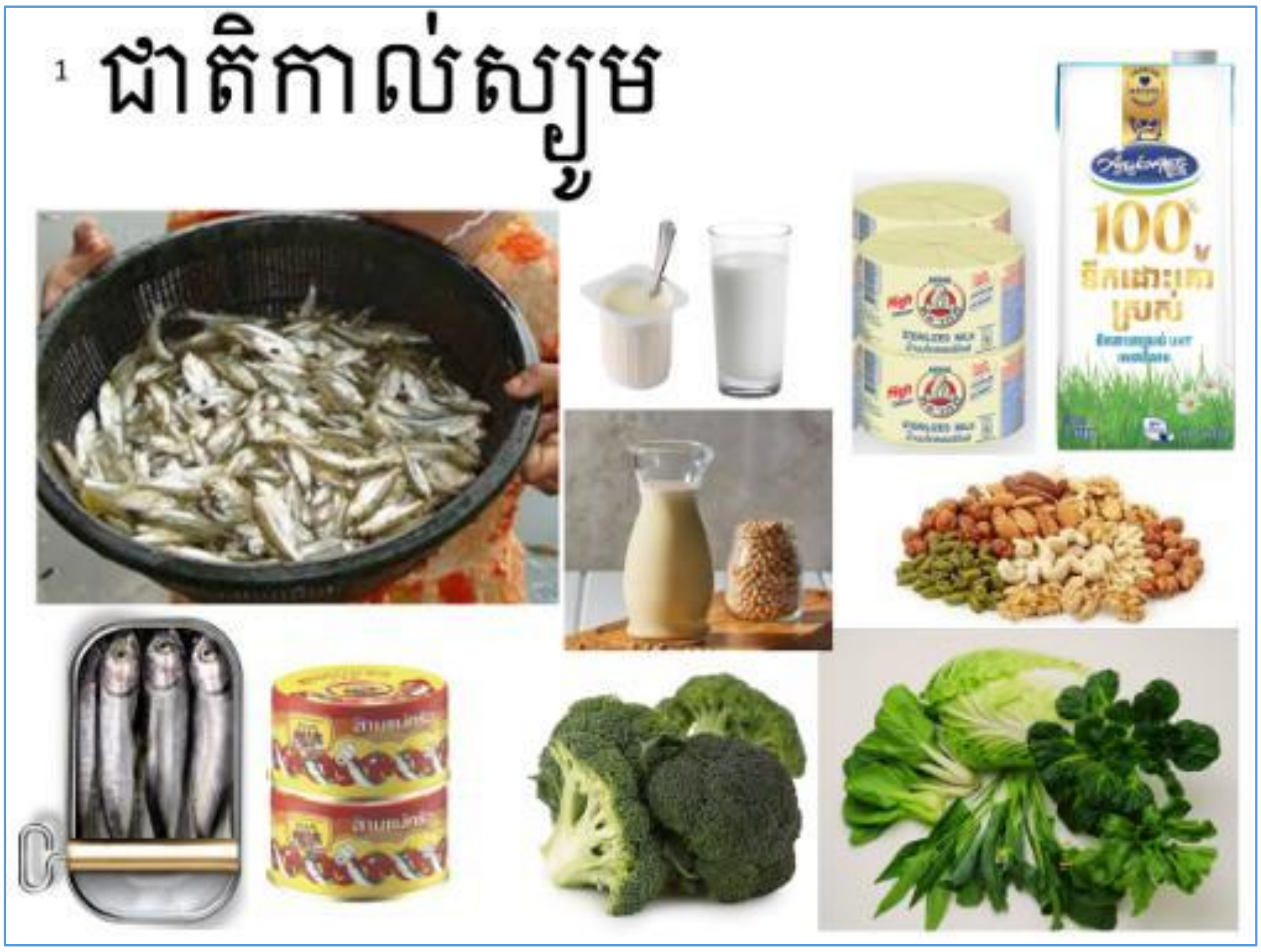

Slide 2: benefits (signs) of adequate calcium

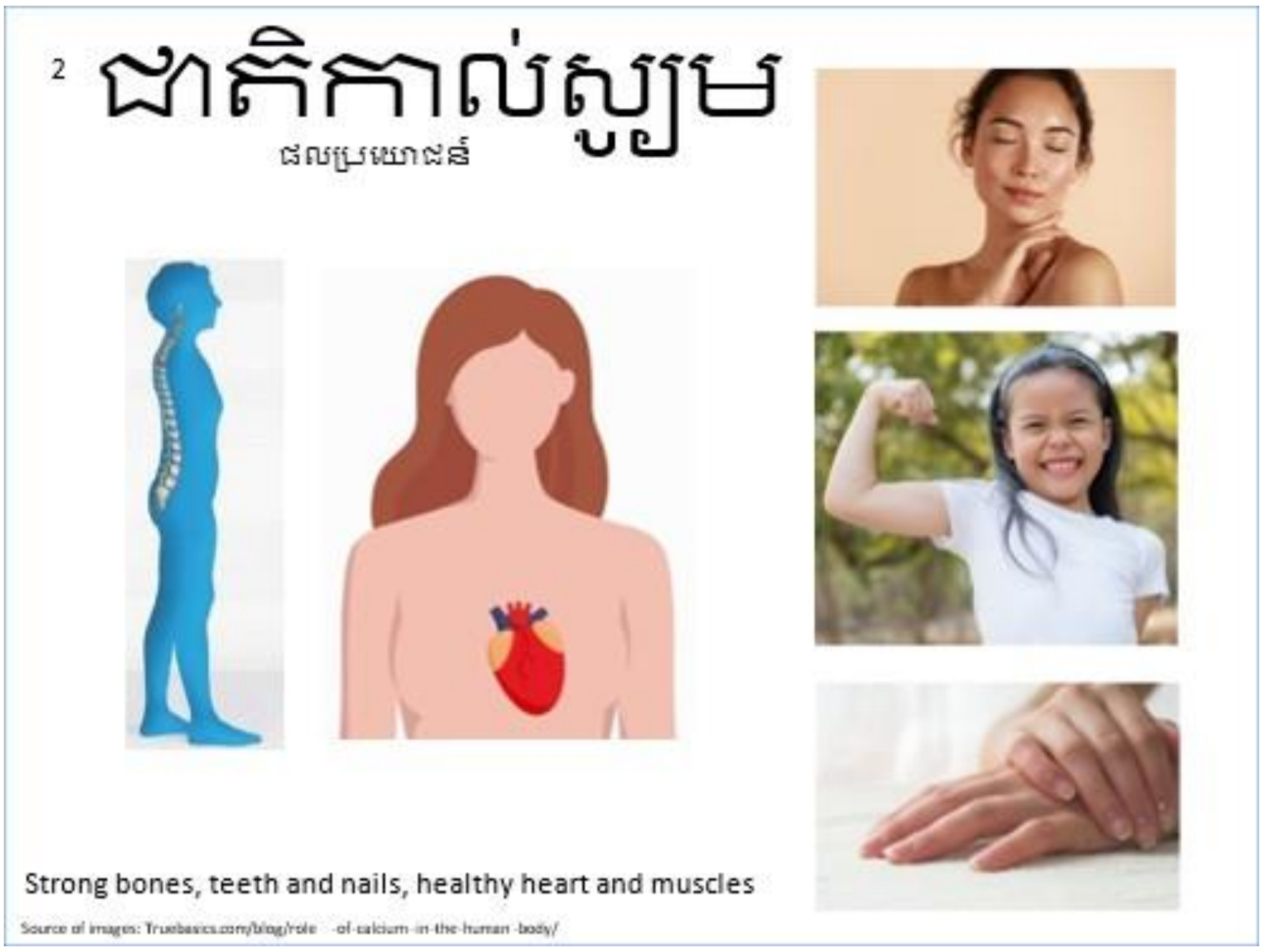

Slide 1: food sources of iron

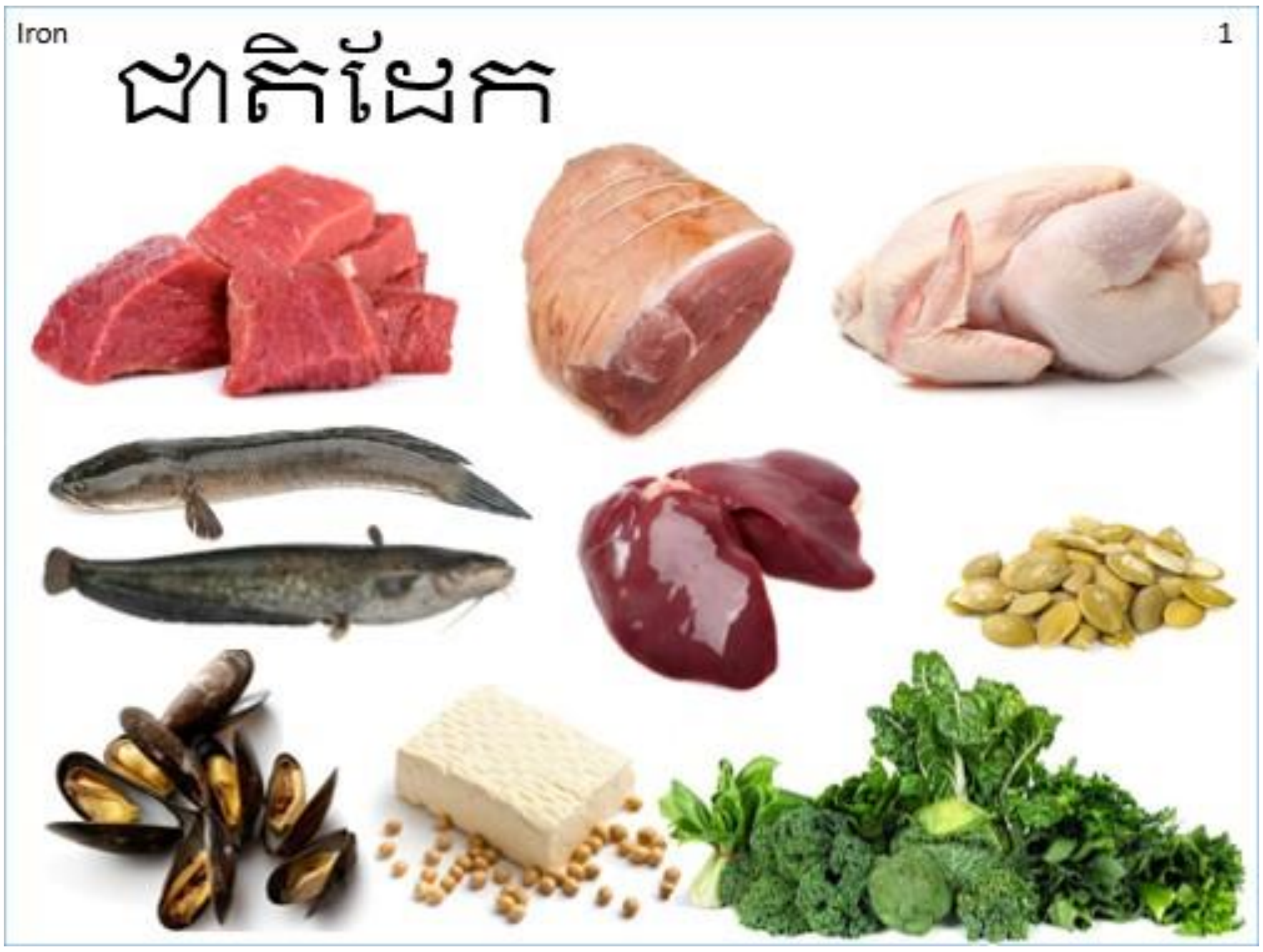

Slide 2: benefits (signs) of adequate iron

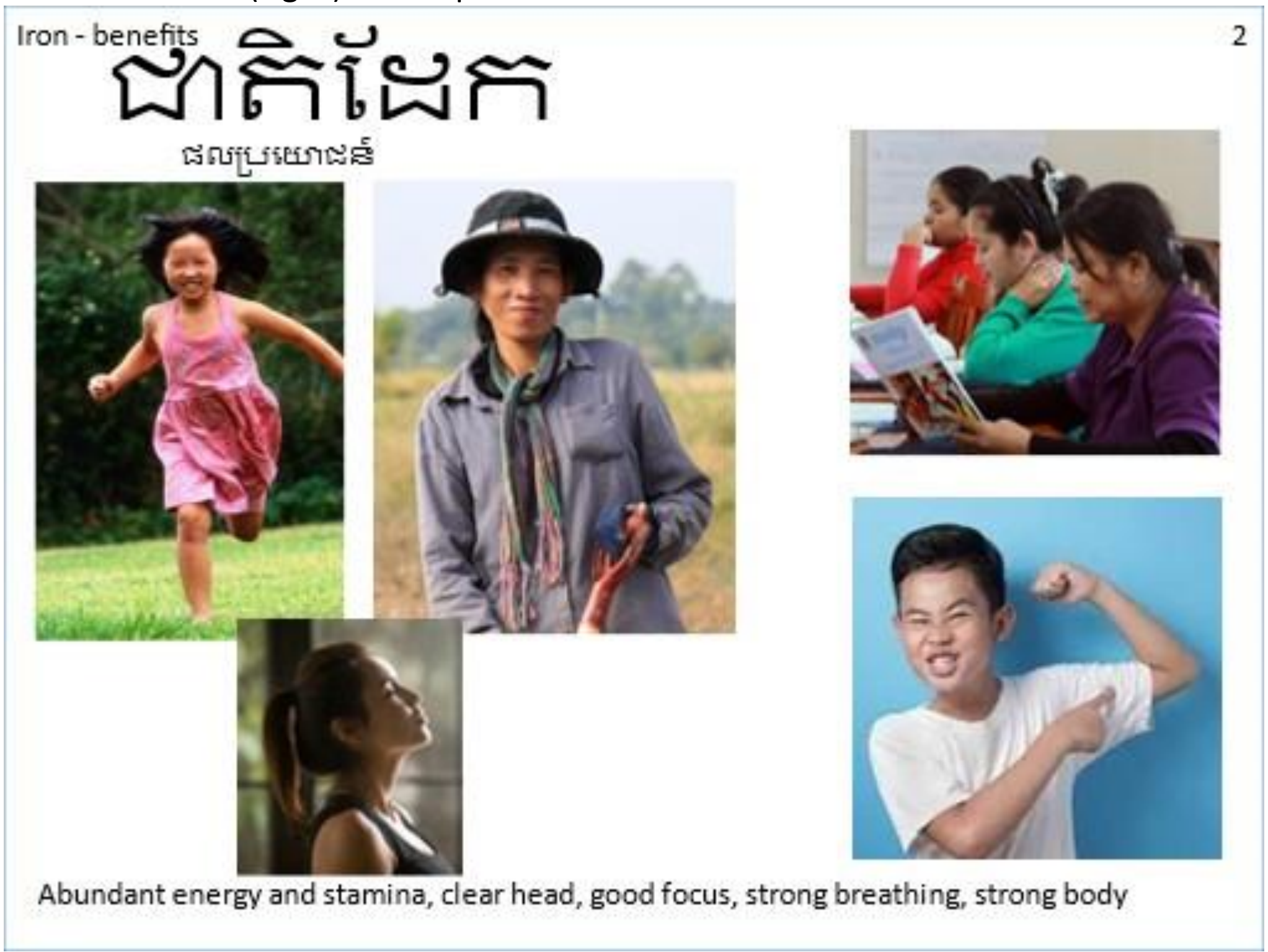

Slide 1: food sources of vitamin A

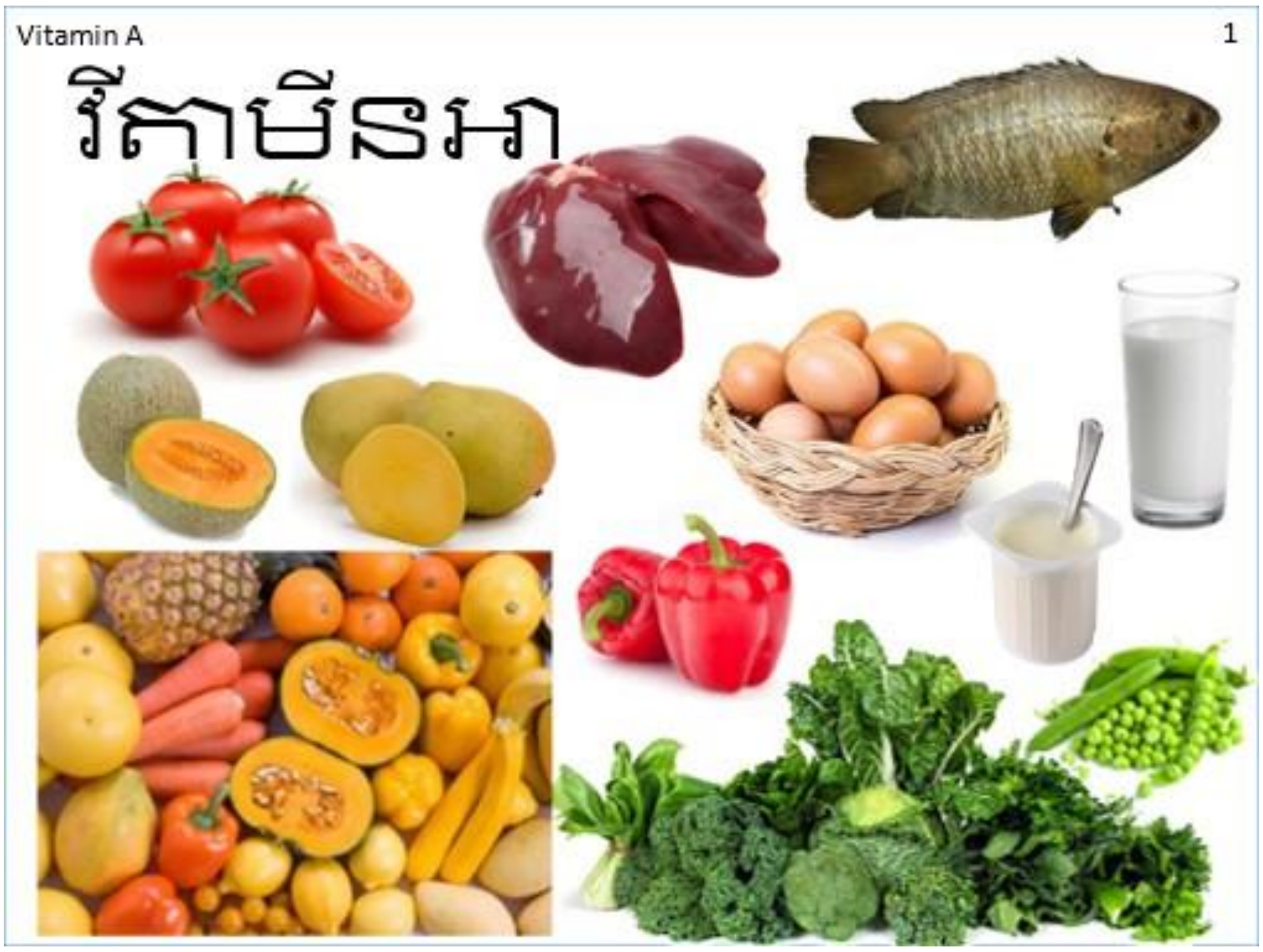

Slide 2: benefits (signs) of adequate vitamin A

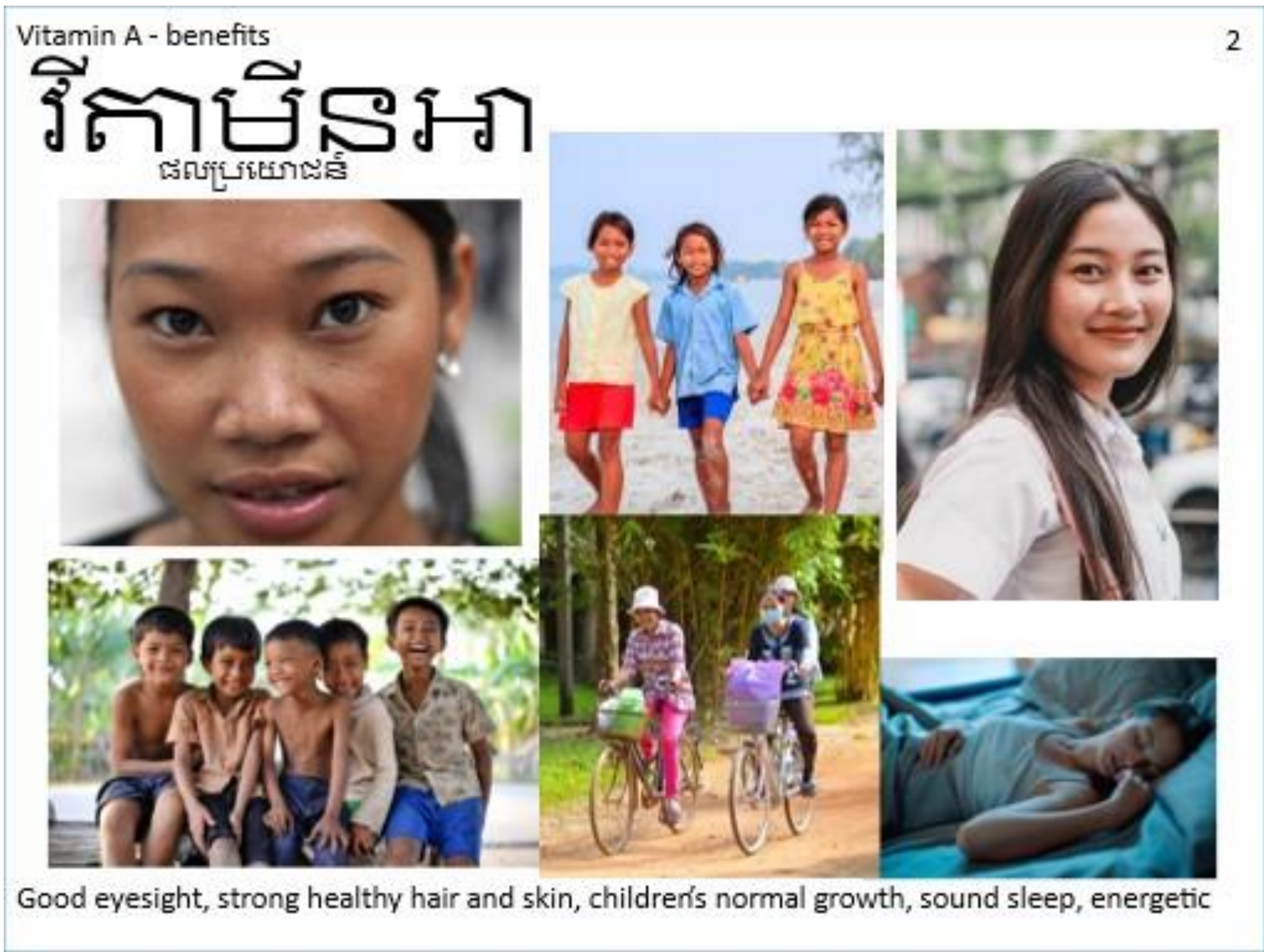

Slide 1: food sources of thiamine

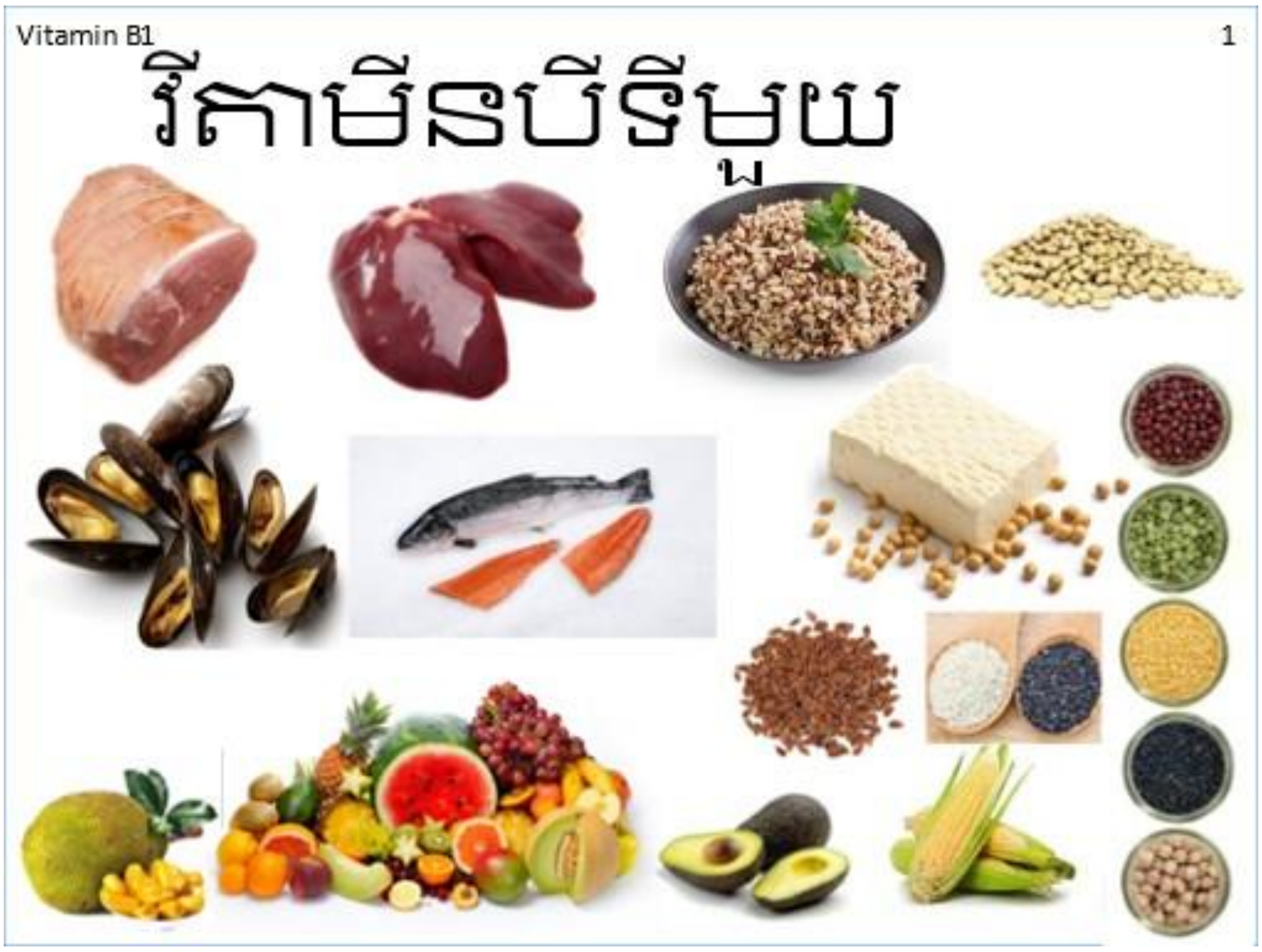

Slide 2: benefits (signs) of adequate thiamine

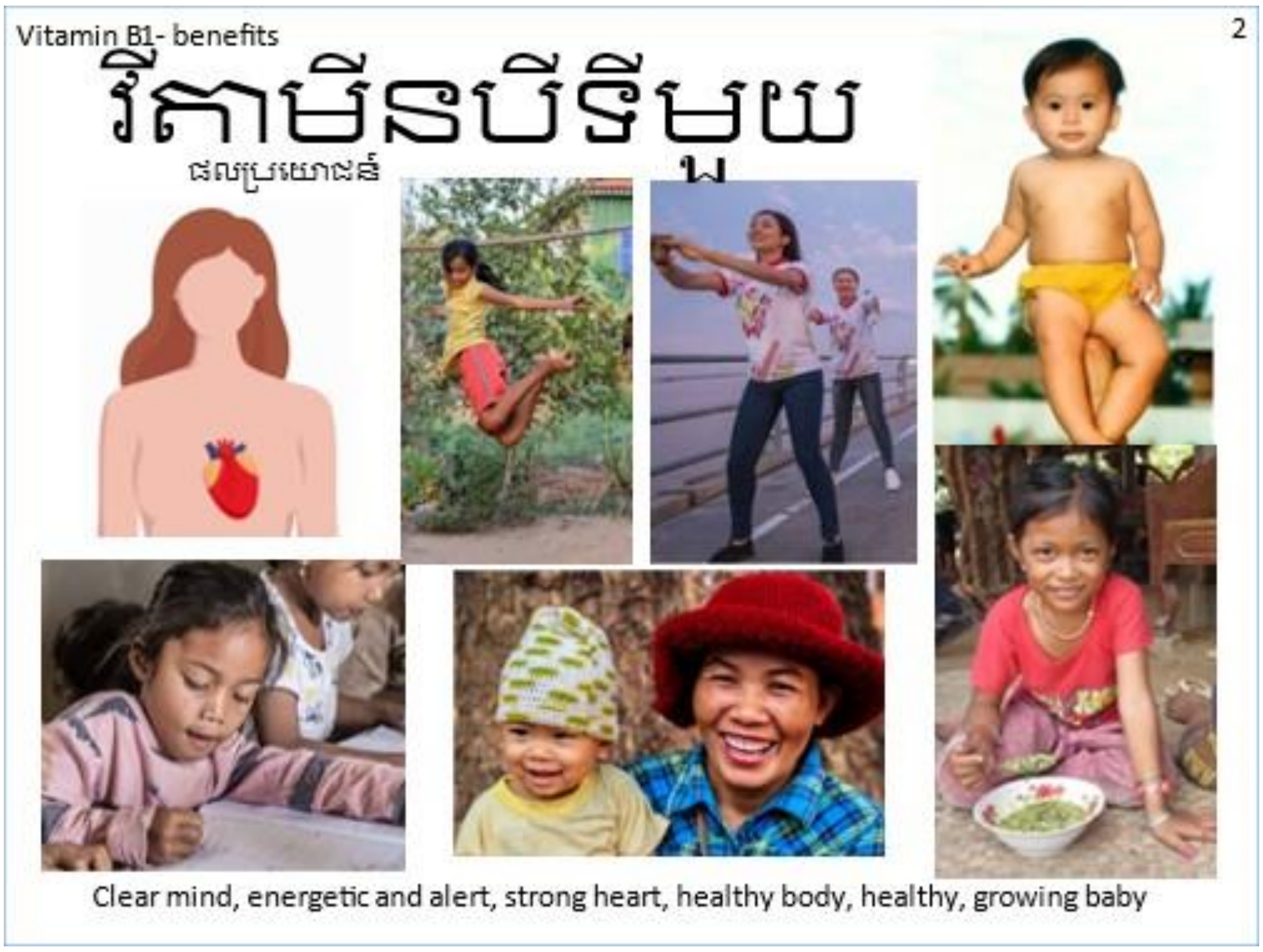

Slide 1: food sources of zinc

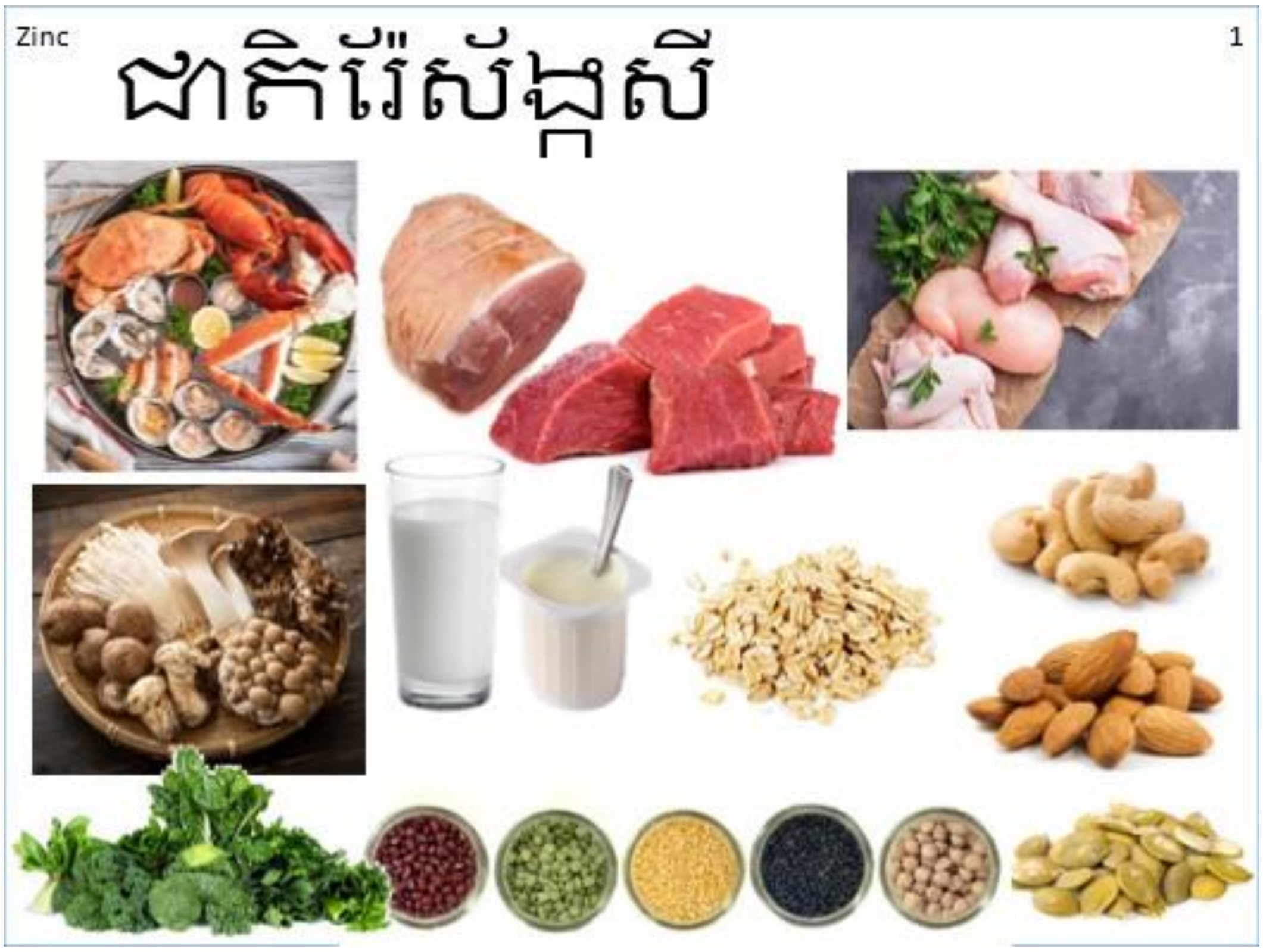

Slide 2: benefits (signs) of adequate zinc

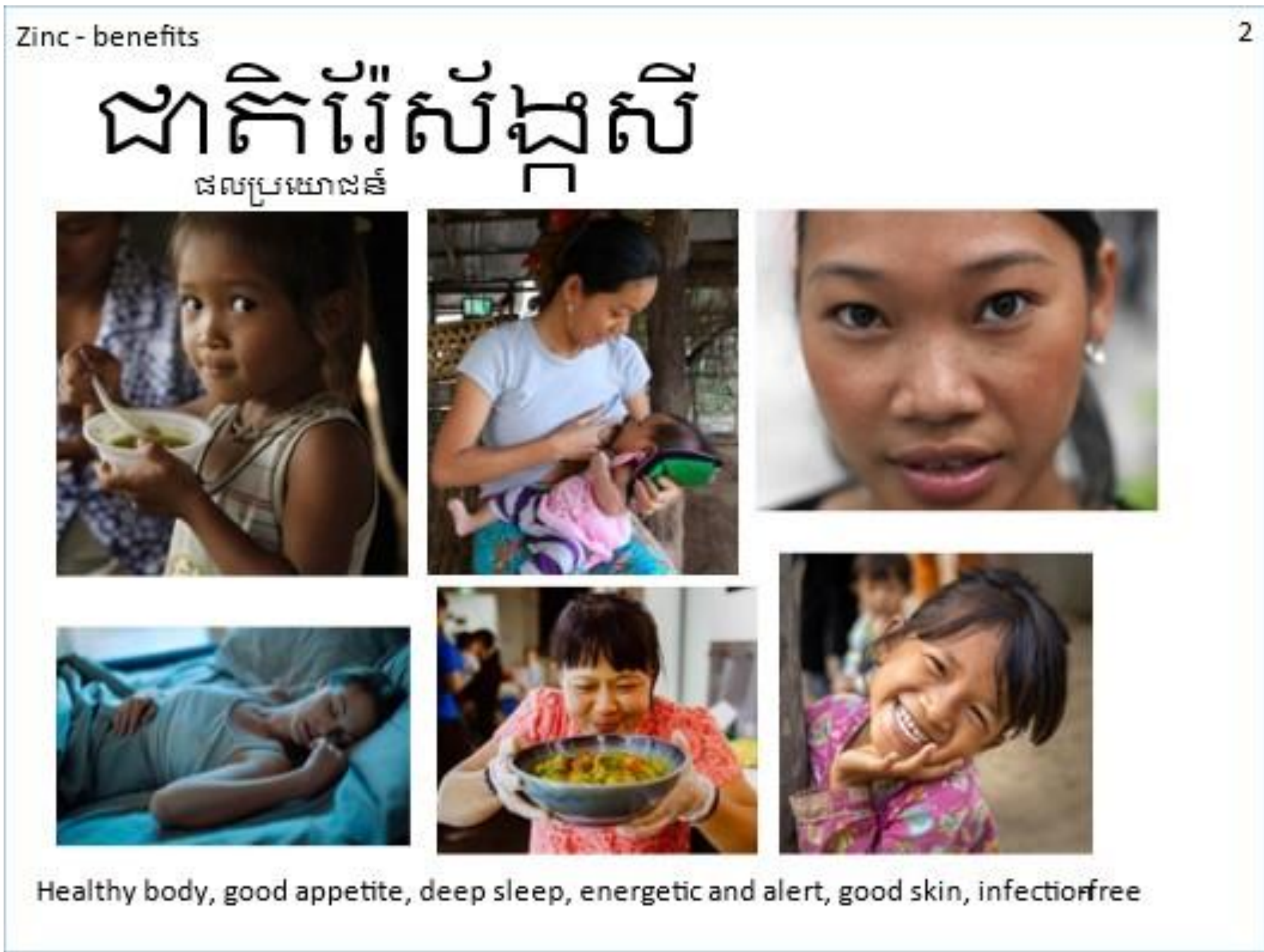

Supplement: Supplementary file 1 [file sj-pdf-1-qch-10.1177_2752535X251374278.pdf]
