# Supplementary material for: Influences on Food Choices of Cambodian Women for Themselves and Their Families
Source: Community Health Equity Res Policy. 2025 Sep 10;47(1):49–61. doi: 10.1177/2752535X251374278 (PMC13499906; doi:10.1177/2752535X251374278)
Supplement: Supplementary file 2 [file sj-pdf-3-qch-10.1177_2752535X251374278.pdf]

Supplementary File 3a: Characteristics of the Cambodian women who participated in the focus groups (n=34)

ID	Location	Age	HH members	Age of HH members	No. HH members	No. children	Marital status	Occupation	Highest school year/ qualification	Market behaviour
P1	Rural	33	husband<br>daughter<br>son<br>daughter	42<br>16<br>12<br>3	4	3	married	farmer/ manual	2	cook meals at home for HH
P2	Rural	29	husband<br>son<br>daughter	35<br>8<br>3	3	2	married	home duties	8	cook meals at home for HH
P3	Rural	46	husband<br>1st child<br>2nd child<br>3rd child<br>4th child<br>5th child<br>6th child	47<br>23<br>21<br>18<br>16<br>13<br>2	7	6	married	farmer/ manual	0	Not stated
P4	Rural	32	husband<br>sister<br>son<br>daughter	37<br>32<br>7<br>3	4	3	married	serve in church	12	go to market; cook meals at home
P5	Rural	40	husband<br>son<br>daughter<br>daughter<br>son	25<br>16<br>9<br>4<br>2	5	4	married	farmer/ manual	7	rarely go to market
P6	Rural	38	husband<br>1st child<br>2nd child<br>3rd child<br>4th child	40<br>17<br>15<br>13<br>5	5	4	married	farmer/ manual<br>home duties	0	rarely go to market
P7	Rural	38	husband<br>1st child<br>2nd child<br>3rd child	37<br>13<br>9<br>3	4	3	married	farmer/ manual<br>home duties	2	go to market; cook meals at home
P8	Rural	32	husband<br>1st child<br>2nd child<br>3rd child	32<br>12<br>3<br>1.5	4	3	married	farmer/ manual<br>home duties	2	go to market; cook meals at home
P9	Rural	38	husband<br>1st child<br>2nd child<br>3rd child<br>4th child<br>5th child	43<br>15<br>13<br>10<br>8<br>5	6	5	married	farmer/ manual<br>home duties	0	go to market; cook meals at home
P10	Rural	24	husband<br>1st child	29<br>4	2	1	married	farmer/ manual<br>home duties	5	don't go to market
P11	Rural	29	husband<br>1st child<br>2nd child	38<br>9<br>4	3	2	married	farmer/ manual	NS	Not stated
P12	Rural	34	husband<br>daughter	?<br>4	2	1	married	Seller	2	go to market; cook meals at home
P13	Rural	35	husband<br>daughter<br>son	?<br>3<br>2	3	2	married	project worker	12	go to market; cook meals at home

ID	Location	Age	HH members	Age of HH members	No. HH members	No. children	Marital status	Occupation	Highest school year/ qualification	Market behaviour
P14	Rural	27	son<br>daughter	5<br>3	2	1	married	teacher	10	go to market; cook meals at home
P15	Rural	31	husband<br>daughter<br>daughter<br>son	43<br>16<br>14<br>5	4	3	married	farmer/ manual	3	go to market; cook meals at home
P16	Rural	40	husband<br>son<br>son<br>daughter<br>daughter	44<br>20<br>17<br>14<br>5	5	4	married	farmer/ manual	0	go to market; cook meals at home
P17	Rural	NS	husband<br>daughter	29<br>2	2	1	married	teacher	diploma	go to market; cook meals at home
P18	Rural	39	husband<br>son<br>daughter<br>daughter<br>daughter	38<br>18<br>19<br>15<br>5	5	4	married	farmer	0	go to market; cook meals at home
P19	Urban	30	husband<br>daughter<br>daughter	35103	3	2	married	home duties	NS	cook meals at home for HH
P20	Urban	38	son<br>daughter<br>son<br>daughter	15<br>11<br>9<br>4	4	4	widowed	home duties	2	cook meals at home for HH
P21	Urban	28	husband<br>daughter	29<br>3	2	1	married	home duties	3	cook meals at home for HH
P22	Urban	31	husband<br>daughter<br>son	32<br>6<br>4	3	2	married	organisation	Bachelor	cook meals at home for HH
P23	Urban	41	husband<br>1st child<br>2nd child<br>3rd child	?<br>16<br>12<br>3	4	3	married	tailor	9	go to market; cook meals at home
P24	Urban	28	husband<br>son	25<br>1	2	1	married	home duties	7	cook meals at home for HH
P25	Urban	35	husband<br>son<br>son	36<br>14<br>5 months	3	2	married	home duties	5	cook meals at home for HH
P26	Urban	NS	husband<br>1st child<br>2nd child	30<br>6<br>2	3	2	married	home duties	8	go to market; cook meals at home
P27	Urban	35	son<br>son	10<br>4	2	2	widowed	home duties	8	cook meals at home for HH
P28	Urban	29	husband<br>1st child<br>2nd child	28<br>5<br>6 months	3	2	married	home duties	NS	Not stated
P29	Urban	38	daughter<br>son	7<br>2	2	2	widowed	organisation	7	cook meals at home for HH
P30	Urban	44	1st child<br>2nd child<br>3rd child	17<br>14<br>4	3	3	widowed	seller	9	cook meals at home for HH

ID	Location	Age	HH members	Age of HH members	No. HH members	No. children	Marital status	Occupation	Highest school year/ qualification	Market behaviour
P31	Urban	41	husband<br>daughter<br>son<br>son	42<br>23<br>19<br>4	4	3	married	home duties	4	cook meals at home for HH
P32	Urban	42	husband<br>daughter<br>son	41<br>6<br>1	3	2	married	organisation	9	cook meals at home for HH
P33	Urban	34	husband<br>1st child<br>2nd child	41<br>7<br>2 months	3	2	married	home duties	1	cook meals at home for HH
P34	Urban	39	husband<br>daughter<br>son<br>son<br>daughter<br>son	40<br>18<br>15<br>12<br>9<br>3	6	5	married	organisation	8	cook meals at home for HH

File 3b: Comparison of rural and urban characteristics of Cambodian women who participated in focus groups (n=34)

	**Rural women** (n=18)	**Urban women** (n=16)
Participant's age	mean: 34.4 years<br>range: 24-46 years	mean: 35.5 years<br>range: 28-44 years
Children's age range	1.5 - 23 years	2 months - 23 years
Number of household members	mean: 3.9 total<br>mean: 2.9 children	mean: 3.1 total<br>mean: 2.4 children
Marital status	18 married<br>0 widowed	12 married<br>4 widowed
Occupation	12 farmers<br>6 home duties<br>5 seller/teacher/other	10 home duties<br>4 organisation<br>2 seller/tailor
Education level reached	5 no school<br>6 primary<br>5 secondary<br>1 tertiary<br>1 not stated	5 primary<br>8 secondary<br>1 tertiary<br>2 not stated
